# An RNA-Seq atlas of gene expression in mouse and rat normal tissues

**DOI:** 10.1038/sdata.2017.185

**Published:** 2017-12-12

**Authors:** Julia F. Söllner, German Leparc, Tobias Hildebrandt, Holger Klein, Leo Thomas, Elia Stupka, Eric Simon

**Affiliations:** 1 Target Discovery, Research Boehringer Ingelheim Pharma GmbH & CoKG, Biberach 88397, Germany; 2 Integrative Transcriptomics, Center for Bioinformatics, University of Tübingen, 72076 Tübingen, Germany; 3 Cardiometabolic Research, Research Boehringer Ingelheim Pharma GmbH & CoKG, Biberach 88397, Germany

**Keywords:** Data publication and archiving, Gene expression, Evolutionary genetics

## Abstract

Gene functionality is closely connected to its expression specificity across tissues and cell types. RNA-Seq is a powerful quantitative tool to explore genome wide expression. The aim of this study is to provide a comprehensive RNA-Seq dataset across the same 13 tissues for mouse and rat, two of the most relevant species for biomedical research. The dataset provides the transcriptome across tissues from three male C57BL6 mice and three male Han Wistar rats. We also describe our bioinformatics pipeline to process and technically validate the data. Principal component analysis shows that tissue samples from both species cluster similarly. We show by comparative genomics that many genes with high sequence identity with respect to their human orthologues also have a highly correlated tissue distribution profile and are in agreement with manually curated literature data for human. In summary, the present study provides a unique resource for comparative genomics and will facilitate the analysis of tissue specificity and cross-species conservation in higher organisms.

## Background & Summary

Biological cells have multiple functions within the body: They may act as small reactors transforming and exchanging energy and organic compounds within their compartments and tissue environment. They transmit or modulate biochemical and physical signals and provide structural integrity. These functions are determined by the abundance and activity of co-expression networks. Despite the progress of protein quantification techniques including mass spectrometry and other methods^1^, there are still major limitations which hamper proteome wide quantification. Factors include sensitivity (i.e., detection of low abundant proteins) and scope (i.e., large proteins, separation of protein complexes and detection of posttranslational modifications^2^). This is reflected by the observation that public repositories of proteomics data are underused by the scientific community^3^ compared to the RNA field. In contrast, RNA resources, for example the Gene Expression Omnibus (GEO) and ArrayExpress, are widely used resources for end users as well as for some powerful public tools like the Expression Atlas (https://www.ebi.ac.uk/gxa/home), or commercial tools like the nextbio BaseSpace Correlation Engine (https://www.nextbio.com), Genevestigator (https://www.genevestigator.com) or Genestack (https://www.genestack.com).

Although DNA microarrays are still widely used, RNA-Seq by next generation sequencing (NGS) is now the technology of choice for ‘transcriptome wide’ gene expression quantification. A wide range of protocols allows RNA-Seq of dissected samples from complex tissues, body fluids, cell-type enriched biosamples and single cells. Depending on the type of RNA preparation (i.e., mRNA, total RNA, etc.), sequencing protocol and sequencing depth, this method allows a full spectrum of transcriptome related read-outs and bioinformatics applications beyond gene expression i.e., inferring strand-, isoform- and sequence variant-specific information which is a unique feature of this technology compared to DNA microarrays or real-time polymerase chain reaction (rPCR) based methods^4^.

A number of important RNA-Seq projects for human tissues have been established which allow in depth exploration of the human transcriptome across a wide range of tissues and cell types^5–7^. However, to our knowledge there is no homogenous RNA-Seq dataset for both mouse and rat. Existing studies that are published rather focus on a single species on specific aspects such as ageing and development^8^, on a few organs^9^ or are based on alternative technologies e.g., genome wide microarrays^10^. Thus a scientist wanting to compare in depth features of genes across species in the same tissue would be only left with the option of performing a meta-analysis across datasets generated in different labs under different conditions.

The present study provides access to a normal tissue gene expression atlas for male C57BL6 mice and male Han Wistar rats. Each tissue atlas is represented by 13 aligned normal tissues (see Table 1). Samples have been dissected from three animals each within a single experiment thus avoiding potential batch effects. Isolated RNA has been sequenced on an Illumina HiSeq2000 with a 50 bp single end read length at a sequencing depth of approximately 20 million reads per sample. The raw data has been processed and analyzed by our automated bioinformatics pipeline which is described in detail in the methods section. Sequenced reads were processed with a mean unique exonic mapping rate of 59% per sample. Within the same species and tissue there is a very high sample-to-sample correlation of the normalized gene expression data. Principal component analysis showed a strong clustering by tissue and a relatively good agreement between mouse and rat tissue specific sample clusters. Consequently, we conclude that the expression variability between samples from the same tissue across different animals is low compared to the expression variability across different tissues from the same animal. We also compared the tissue specific expression patterns between mouse and rat for a subset of genes with high sequence conservation with respect to human. Accordingly, many highly conserved genes also have highly correlated gene expression patterns. In addition, the observed tissue specificity of most of these genes was also confirmed by manually curated literature data provided by the UniProt/Swiss-Prot database.

## Methods

### Animal study

Male Wistar Han rats (Crl:WI(Han)) and male BL/6J mice (C57BL/6J) were obtained from Charles River Laboratories (Germany). Experimental protocols concerning the use of laboratory animals were reviewed by a German Federal Ethics Committee and approved by German governmental authorities. Animals were housed in groups of three on a 12-h light/dark cycle and fed ad libitum a standard pelleted rodent diet (Diet No. 3438, Provimi Kliba Switzerland) with free access to water. Rats with a body weight of 160–180 g and mice at the age of 7–8 weeks were used for tissue sampling. Animals (*n*=3 for each species) were sacrificed thereafter by intraperitoneal injection of pentobarbital (rats) or cervical dislocation (mice) and tissues (esophagus, stomach, duodenum, jejunum, ileum, colon, pancreas, liver, thymus, kidney, heart, brain, quadriceps muscle) were harvested and transferred immediately to RNA Later at 4 °C.

### RNA extraction, illumina library preparation and sequencing

Total RNAs were individually extracted using the Ambion Magmax™-96 total RNA isolation kit (Life Sciences) according to the manufacturer’s instructions. Briefly, 5 mg of tissue was placed in the lysis solution and homogenized in Qiagen Tissuelyzer™ for a period of 30 s. Nucleic acids were captured onto magnetic beads, washed and treated with DNase. Total RNA was then eluted in 50 μl elution buffer. RNA quality and concentration was measured using an RNA Pico chip on an Agilent Bioanalyzer.

The Sequencing library preparation has been done using 200 ng of total RNA input with the TrueSeq RNA Sample Prep Kit v2-Set B (RS-122–2002, Illumina Inc, San Diego, CA) producing a 275 bp fragment including adapters in average size. In the final step before sequencing, eight individual libraries were normalized and pooled together using the adapter indices supplied by the manufacturer. Pooled libraries have then been clustered on the cBot Instrument from Illumina using the TruSeq SR Cluster Kit v3—cBot—HS(GD-401–3001, Illumina Inc, San Diego, CA) sequencing was then performed as 50 bp, single reads and 7 bases index read on an Illumina HiSeq2000 instrument using the TruSeq SBS Kit HS- v3 (50-cycle) (FC-401–3002, Illumina Inc, San Diego, CA).

### mRNA-Seq bioinformatics analysis

The processing pipeline is described in detail below. One sample could not be processed due to technical issues (mouse_11_heart). For all remaining samples, RNA-Seq reads from rat and mouse samples were aligned to the rat and mouse genomes respectively using the STAR Aligner v2.5.2a^11^ with their corresponding Ensembl 84 reference genomes (http://www.ensembl.org). Sequenced read quality was checked with FastQC v0.11.2 (http://www.bioinformatics.babraham.ac.uk/projects/fastqc/) and alignment quality metrics were calculated using the RNASeQC v1.18^12^. Following read alignment, duplication rates of the RNA-Seq samples were computed with bamUtil v1.0.11 to mark duplicate reads and the dupRadar v1.4 Bioconductor R package for assessment^13^. The gene expression profiles were quantified using Cufflinks software version 2.2.1^14^ to get the Reads Per Kilobase of transcript per Million mapped reads (RPKM) as well as read counts from the feature counts software package^15^. The matrix of read counts and the design file were imported to R, normalization factors calculated using trimmed mean of M-values (TMM) and subsequently voom normalized, before subjected to downstream descriptive statistics analysis.

### Step-by-step mRNA-Seq pipeline

Before running the execution steps mentioned above, one has to prepare target organism alignment indices for the STAR aligner. For mouse this is done as follows:

STAR --runMode genomeGenerate \

--genomeDir mouse84.STARIndex/ \

--genomeFastaFiles Mus_musculus.GRCm38.dna.primary_assembly.fa \

--sjdbGTFfile Mus_musculus.GRCm38.84.gtf \

--sjdbOverhang 49 \

--runThreadN 16

For rat this has to be adopted accordingly. After the genome index is prepared, all samples from each species are processed individually. In all subsequent commands <sample_id> corresponds to the sample name (for example 199_1 for the first mouse sample from the pancreas).

Make a sample output directory, where all the outputs from each step will be stored:

mkdir <sample_id>

Check sequenced read qualities with FastQC v0.11.2:

fastqc --outdir=<sample_id>/ <sample_id> &> <sample_id>/<sample_id>.fastqc.log

Align reads using the STAR aligner v2.5.2a:

STAR --genomeDir mouse84.STARIndex/ \

--readFilesIn <sample_id>.fastq.gz \

--outFileNamePrefix <sample_id>/<sample_id>.fastq.gz. \

--runThreadN 8 \

--limitBAMsortRAM 60000000000 \

--outSAMattrRGline ID:<sample_id>.fastq.gz SM:<sample_id>.fastq.gz \

--outBAMsortingThreadN 8 \

--outSAMtype BAM SortedByCoordinate \

--outSAMunmapped Within \

--outSAMstrandField intronMotif \

--readFilesCommand zcat \

--chimSegmentMin 20 \

--genomeLoad NoSharedMemory

Create BAM file index (*.bai) using samtools v0.1.18:

samtools index <sample_id>/<sample_id>.fastq.gz.Aligned.sortedByCoord.out.bam

Mark Duplicates using BamUtils v1.0.11 ‘dedup’ step:

bam dedup --in <sample_id>/<sample_id>.fastq.gz.Aligned.sortedByCoord.out.bam \

--log <sample_id>/<sample_id>.fastq.gz.Aligned.out.dupmark.log \

--out <sample_id>/<sample_id>.fastq.gz.Aligned.out.dupmark.bam \

--noPhoneHome

samtools index <sample_id>/<sample_id>.fastq.gz.Aligned.out.dupmark.bam

Run DupRadar v1.4 on the duplicate marked bam:

mkdir <sample_id>/dupradar

dupRadar.sh --bam=<sample_id>/<sample_id>.fastq.gz.Aligned.out.dupmark.bam \

--gtf=Mus_musculus.GRCm38.84.gtf \

--stranded=no \

--paired=no \

--outdir=<sample_id>/dupradar \

--threads=16

Gene/Transcript quantification with Cufflinks v.2.2.1 to get RPKMs:

cufflinks -u -p 8 -o <sample_id>/cufflinks \

--max-bundle-frags 1000000000 \

--no-effective-length-correction \

--compatible-hits-norm \

-G Mus_musculus.GRCm38.84.gtf \

<sample_id>/<sample_id>.fastq.gz.out.dupmark.bam

Run featureCounts to generate read counts:

featureCounts -a Mus_musculus.GRCm38.84.gtf \

-o <sample_id>/<sample_id>.fastq.gz.featureCounts.ensembl.txt \

-T 3 <sample_id>/<sample_id>.fastq.gz.Aligned.out.markdup.bam

RNA quality control:

java -Xmx20g -jar RNA-SeQC_v1.1.8.jar \

-t Mus_musculus.GRCm38.84.gtf \

-r Mus_musculus.GRCm38.dna.primary_assembly.fa \

-o <sample_id>/rnaqc -singleEnd -ttype 2 \

-s ‘<sample_id>|<sample_id>.fastq.gz.Aligned.out.dupmark.bam|Notes’

All per sample output is finally merged into the read count (<species>_counts.txt), RPKM (<species>_rpkm.txt), and technical QC (<species>_rnaqc.txt) tabular output files. The graphics in Supplementary S1 summarize the results of RNA quality control for all mouse and rat samples.

### Downstream analysis of mouse and Rat RNA-Seq datasets

**Figure 1:**
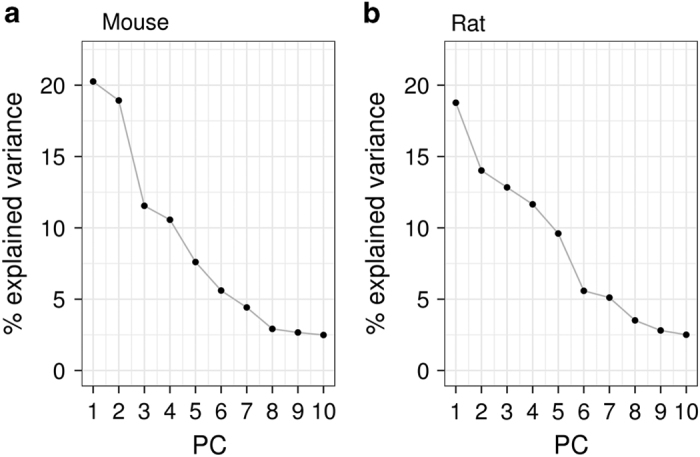
Explained variance by principal components. The line plots show percentage of explained variance for the first ten principal components for the mouse (**a**) and rat (**b**) samples.

**Figure 2:**
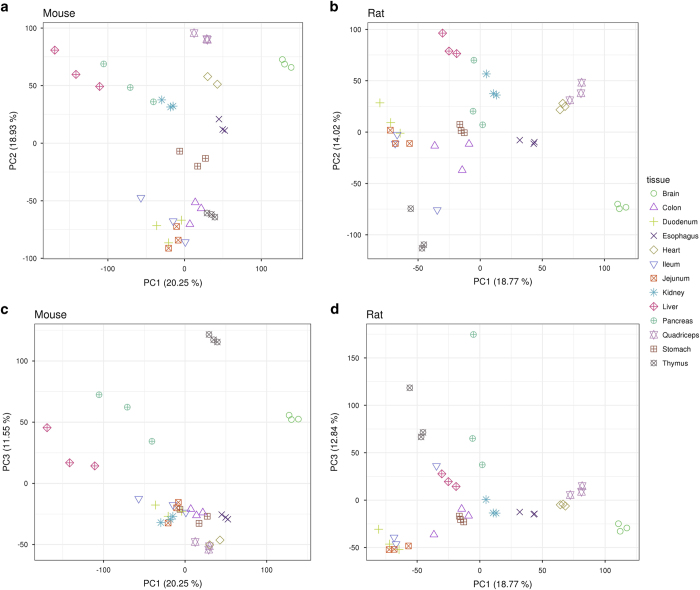
Principal component analyses (PCA). Scatter plot of the dimensions^24^ PC1 versus PC2 and PC1 versus PC3 (mouse: **a**,**c**; rat: **b**,**d**). Samples are colored by tissue and the numbers in brackets correspond to the proportion of variance explained by the respective principal component.

**Figure 3:**
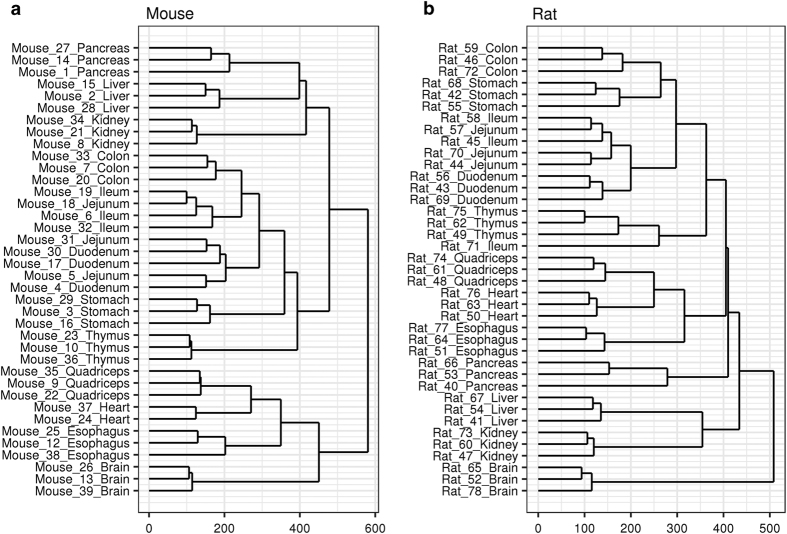
Hierarchical clustering. Hierarchical clustering of mouse (**a**) and rat samples (**b**). Dendrograms visualizing the result of hierarchical clustering based on voom-transformed log(counts per million). Euclidean distance between samples and the complete linkage method were used for clustering.

**Figure 4:**
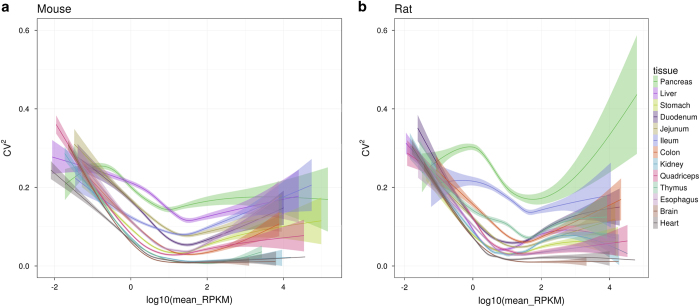
Expression variability across tissues. Squared coefficient of variation for mouse (**a**) and rat (**b**) genes versus their log10(mean_RPKM) colored by tissue to visualize intra- and inter-tissue variation^17^. The smoothing function used is gam (generalized additive models with integrated smoothness estimation), thus standard errors based on the posterior distribution of the model coefficients are shown.

After preprocessing the raw mouse and rat data independently, the subsequent downstream analysis has been applied to the merged data tables using the R-code provided in Supplementary S2. In brief, both datasets were imported into a common working environment and several descriptive analyses were performed. A principal component analysis (Figs 1 and 2) and hierarchical clustering (Fig. 3) were done on limma^16^ voom-transformed log(counts per million). Intra- and inter-tissue variation was assessed based on RPKM expression values (Fig. 4). For the orthology analyses (Fig. 4) both datasets were limited to protein coding genes which have a one-to-one homology relationship to human genes and between mouse and rat and vice versa. The homology information and gene biotype annotations were obtained from Ensembl Version 84.

## Data Records

A complete list of the 77 tissue samples with sample ids is given in Table 1. The fastq files with the sequencing raw data and all metadata needed to run the R code have been deposited to ArrayExpress (Data Citation 1).

## Technical Validation

### Data QC from the pipeline with PCA

Total number of reads varied between 20 and 30 million per sample. The 50 bp single-end reads were processed with a unique exonic mapping rate of 67% for mouse and 52% for rat. The difference of 15% between the two species probably reflects the level of annotation and curation completeness of both reference genomes. For technical validation we analyzed both datasets independently at the sample level by principal component analysis, hierarchical clustering and by investigating the distribution of expression values. We also compared the overall expression variability and the transcriptomic profiles of both species and used sequence homology information to link the data to human.

The high dimensionality of the datasets is reflected by the results from the principal component analysis. Although most variance is explained by the first three dimensions there is only a moderate decline in the fraction of explained variance at higher dimensions in both datasets (see also Fig. 1). In the first two dimensions, PC1 and PC2, one can observe a consistent clustering of the samples by tissue (see Fig. 2a,b). In both species, brain samples form the most distinct cluster along PC1. A second cluster is composed of the samples from heart and skeletal muscle (top right) followed by central clusters of liver, pancreas, kidney, stomach and esophagus samples. All samples from the gastrointestinal (GI) tract (i.e., duodenum, jejunum, ileum and colon) and from thymus cluster in the lower part of the two plots. In PC1 versus PC3 (see Fig. 2c,d). Thymus and pancreas samples show the strongest separation from all other tissues.

The predominance of inter-tissue versus inter-animal variance is also confirmed by hierarchical clustering (see Fig. 3). Only samples from the GI tract, specifically the small intestine, show a mixed clustering with respect to their origin from the gut (duodenum, jejunum and ileum). In mouse, the samples from the upper small intestine (duodenum and jejunum) are mixed up and for both species one sample from the jejunum ends up in the corresponding ileum cluster. However, all GI tract samples, including those from stomach, form a supercluster in each species which is separated from the remaining tissue clusters and has thymus as closest neighboring tissue cluster.

### Expression variability across tissues

The intra-tissue variability of gene expression has been investigated by comparing the squared coefficient of variation (standard deviation divided by mean) versus the mean log10 tissue RPKM (see also CummeRbund plots^17^ in Fig. 4). This visualization reveals some common features for mouse and rat. The variation per tissue is relatively high at low and at high mean expression values. For almost all tissues, the mean squared coefficient of variation is decreasing with increasing absolute expression to a minimum value between 0.05 and 0.2 in the interval between 1 and 4 mean RPKM. At lower and at higher mean expression values also the spread of the variation coefficient per tissue is increasing. The order of tissues with highest versus lowest variation is mostly conserved over the dynamic range of absolute expression levels and also similar between mouse and rat, i.e., with pancreas and ileum corresponding to the two tissues with largest variation at higher expression values. However, there is a somewhat lower absolute variance observed for mouse versus rat tissue samples and in rat there is a more pronounced difference between pancreas and ileum and the other tissues.

The tissue samples investigated in the present study correspond to dissected samples from complex organs. Consequently, the gene expression signal from each gene and sample is superimposed by signals that originate from the individual cell types which make up the corresponding organ. Eventually this might explain the observed inverse bell shape distribution of the variation coefficient versus absolute expression. Some cell types express a significant amount of very specific genes because this is an inherent feature of their function. The pancreas, for example, is composed of functionally different cell types implying distinct sets of highly active genes. Insulin is exclusively expressed by beta cells at very high levels. However, pancreatic beta cells only represent a minor fraction of the whole pancreas. Consequently, bulk samples from the pancreas contain a variable composition of different cell types which will contribute to a high expression variability of cell type specific genes like Insulin. INS1 gene expression, for example, shows a relatively wide spread of 389.7, 1449.0 and 4020.8 RPKM in the three rat samples of the present study. Although this is a very strong signal (there are less than 50 genes with a higher median expression in the pancreas samples) it is at least two orders of magnitude lower compared to levels in isolated pancreatic islets (see e.g., mouse islet data from^18^). Consequently, cell type specific dissection, purification and sequencing protocols can largely improve our understanding of cell type specific expression.

In general, the results from the present study are in line with other large RNA-Seq datasets showing that the inter-tissue variability of gene expression is much larger compared to gene expression variability across individuals^19^. An additional argument along this line is the fact that we are looking at individual animals from inbred rodent strains which exhibit a low genetic variability by nature. Thus, we assume that a single controlled experiment on expression in normal tissues at steady state conditions gives a reasonably good estimate of genome wide tissue specific expression at a relatively small sample size.

### Mouse-to-rat correlation

**Figure 5:**
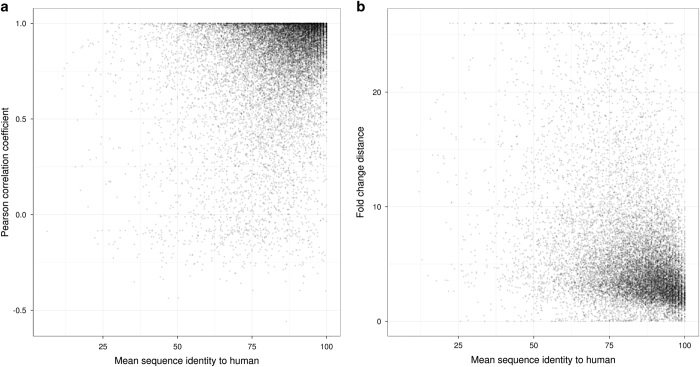
Tissue specific expression versus gene sequence conservation. Mean sequence identity of mouse and rat to their human orthologue versus Pearson’s correlation coefficient (**a**) and fold change distance between rodent gene expression profiles (**b**).

For further technical validation we compared the tissue specific expression between the mouse and rat data in the context of sequence conservation assuming that highly conserved genes also exhibit higher correlation of tissue specificity. Figure 5a shows a scatter plot of the gene-wise correlation coefficients between mouse and rat expression profiles in relation to the mean protein sequence identity to the human gene product. Although there are some genes with low sequence identity and high correlation (and vice versa), the majority of all ~17,000 protein coding genes with a 1:1 homology relationship in mouse versus rat versus human cluster at high correlation coefficients and high sequence conservation. As an aside, there are only a few hundred genes with a negative correlation coefficient. We have investigated some of those genes more in detail. In many of these cases, the expression in one species is quite ubiquitous whereas it is very low or absent in the second species. In most other cases, the expression is restricted to a few tissues but different ones in mouse compared to rat. More in-depth investigations of these differences might help to improve the genome annotations for both rodent strains and to improve our understanding of gene expression variability. However, these aspects are beyond the scope of the present paper.

As an alternative approach for species comparison we normalized the mean gene expression in each tissue by the mean expression in all other tissues and refer to this relative gene expression hereafter as a measure of tissue specificity. For a pair of orthologous genes from mouse and rat we then calculated the Manhattan distance of the two tissue specificity vectors as a measure for tissue specificity similarity between the two species. As shown in Fig. 5b, most genes with a small tissue specificity distance correspond to genes with a high sequence identity with respect to human (i.e., conserved genes). In contrast, the pairwise tissue specificity distance of orthologous genes strongly increases for genes with low sequence conservation in human.

### Inferring conserved genes

By combining the two approaches described in the previous section we selected ultra-conserved genes (sequence identity versus human≥90%) which are highly correlated (r_p_≥0.9) for further examination (see Supplementary S3). Gene set enrichment at MSigDB^20^ (GSE) using canonical pathways from BIOCARTA, KEGG, and REACTOME revealed a highly significant enrichment of genes involved in transcript splicing and RNA processing (FDR<10^−15^) as well as neuronal genes (FDR<10^−7^) and genes involved in the immune system (FDR<10^−7^). Although those gene categories are also found enriched in studies of evolutionary divergence^21^ one should keep in mind that the underlying biological processes are essential for development and homeostasis. Thus, mutations observed in the corresponding human genes are often pathologic^22,23^. Consequently, there is a strong enrichment of genes involved in the immune system (FDR<10^−52^) and neuronal genes (FDR<10^−36^) among the set of known human disease genes according to the Online Mendelian Inheritance in Man (OMIM).

**Figure 6:**
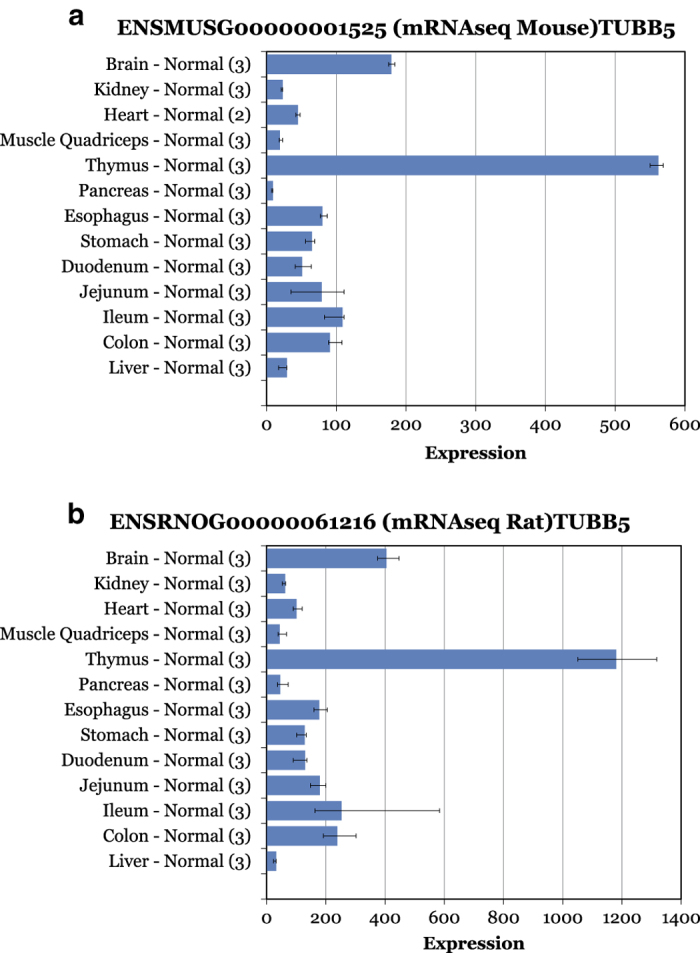
Electronic northern blot of the super-conserved gene Tubb5. Expression numbers on the X-axis correspond to RPKM values for mouse (**a**) and rat (**b**) samples. The size of the blue bar and lower and upper error bars correspond to the mean, min and max value in the corresponding tissue group, respectively. Numbers in brackets correspond to numbers of replicates per group.

To further evaluate the observed expression of these genes we compiled curated information on human tissue specificity from UniProtKB/Swiss-Prot, the manually curated subset of UniProtKB. Table 2 shows the results for the top 20 genes where this information is available. As a first observation there are very high correlation coefficients between mouse and rat expression profiles and the tissue with the highest expression is identical for all pairs of genes. Interestingly, most of these ultra-conserved genes are ubiquitously expressed and / or have the highest expression in brain or thymus. The similarity between the rat and mouse expression profiles for those genes is striking as shown exemplified for TUBB in Fig. 6. The observed distribution in mouse and rat shows a ubiquitous tissue profile with the highest expression in thymus, followed by brain. Furthermore, this pattern matches the manually curated tissue specificity for the corresponding human homolog protein entry (Data Citation 2, see also other examples listed in Table 2).

One should bear in mind that the abundance of a transcript is not always predictive for the abundance of the corresponding protein and there are a number of studies which have investigated this in detail^5^. However, according to the central dogma in molecular biology, the information on a coding gene is transferred from DNA to mRNA and from mRNAs to Protein. Under steady-state conditions in normal tissues, the mean abundance of a protein should correlate well with the mean abundance of the corresponding mRNA transcript, but it is beyond the scope of the present study to investigate possible exceptions from this rule.

Despite the emerging attention of the academic community to non-coding RNAs we focused on mRNA for the present study, because the described RNA and sequencing protocol as well as the data analysis pipeline are optimized for protein coding genes.

In summary, the present study provides access to genome wide gene expression data across complex organs from C57BL6 mice and Han Wistar rats which belong to the most relevant animal model species for biomedical research. Technical quality control and data analysis show a consistent pattern of sample clustering and intra-versus-inter tissue variation of gene expression. We also demonstrate that the present dataset can facilitate a better understanding and awareness of gene function across different species. Comparative genomics and transcriptomics may also help to translate preclinical data into human by confirming cross-species conservation of drug target genes or—the other way round—by predicting potential issues with species selectivity.

## Usage Notes

The R source code to perform the data analysis and to generate the figures of the manuscript is provided in Supplementary S2.

## Additional information

**How to cite this article:** Söllner, J. F. *et al.* An RNA-Seq atlas of gene expression in mouse and rat normal tissues. *Sci. Data* 4:170185 doi: 10.1038/sdata.2017.185 (2017).

**Publisher’s note:** Springer Nature remains neutral with regard to jurisdictional claims in published maps and institutional affiliations.

## Supplementary Material



Supplementary S1

Supplementary S2

Supplementary S3

## Figures and Tables

**Table 1:** Tissues and corresponding mouse and rat sample ids.

**Tissue**	**N Male BL/6J mice (C57BL/6J)**	**N Male Wistar Han rats (Crl:WI(Han))**
Brain	13, 26, 39	52, 65, 78
Kidney	8, 21, 34	47, 60, 73
Heart	24, 37	50, 63, 76
Thymus	10, 23, 36	49, 62, 75
Pancreas	1, 14, 27	40, 53, 66
Esophagus	12, 25, 38	51, 64, 77
Stomach	3, 16, 29	42, 55, 68
Duodenum	4, 17, 30	43, 56, 69
Jejunum	5, 18, 31	44, 57, 70
Ileum	6, 19, 32	45, 58, 71
Colon	7, 20, 33	46, 59, 72
Liver	2, 15, 28	41, 54, 67
Muscle Quadriceps	9, 22, 35	48, 61, 74
**Total**	**38**	**39**
All data files are available at Data Citation 1.		

**Table 2:** Top20 validated super-conserved genes.

**Gene**	**Max Tissue Mouse, Rat**	**Human tissue specificity according to UniProtKB/Swiss-Prot**
PAFAH1B2	Brain, Brain	Ubiquitous
TRA2B	Thymus, Thymus	Highest expression in heart, skeletal muscle and pancreas
TUBB	Thymus, Thymus	Ubiquitously expressed with highest levels in spleen, thymus and immature brain
DPYSL2	Brain, Brain	Ubiquitous
SNRNP200	Thymus, Thymus	Widely expressed
RAB3A	Brain, Brain	Specifically expressed in brain
ARL8A	Brain, Brain	Ubiquitously expressed
SF3A3	Thymus, Thymus	Ubiquitous
UBE2Q1	Thymus, Thymus	Widely expressed
MTPN	Brain, Brain	Ubiquitous
YWHAG	Brain, Brain	Highly expressed in brain, skeletal muscle, and heart
DERL2	Liver, Liver	Ubiquitous
YWHAQ	Brain, Brain	Abundantly expressed in brain, heart and pancreas, and at lower levels in kidney and placenta
FGF9	Brain, Brain	Glial cells
PTBP2	Brain, Brain	Mainly expressed in brain although also detected in other tissues like heart and skeletal muscle
MAGOH	Thymus, Thymus	Ubiquitous
CDC42SE2	Thymus, Thymus	Widely expressed
HNRNPH1	Thymus, Thymus	Expressed ubiquitously
DYRK1A	Thymus, Thymus	Ubiquitous
RUVBL2	Thymus, Thymus	Ubiquitously expressed
Genes were selected based on high correlation of tissue distribution, high sequence conservation versus human and the availability of curated tissue specificity information from the UniProt/Swissprot database.		
