# Supplementary material for: An RNA-Seq atlas of gene expression in mouse and rat normal tissues
Source: Sci Data. 2017 Dec 12;4:170185. doi: 10.1038/sdata.2017.185 (PMC5726313; doi:10.1038/sdata.2017.185)
Supplement: Supplementary S2 [file sdata2017185-s3.pdf]

NGS RNA-Seq Technical QC metrics for "An RNA-Seq atlas of gene expression in mouse and rat normal tissues"

Julia F. Söllner, Germán Leparc, Tobias Hildebrandt, Holger Klein, Leo Thomas, Elia Stupka and Eric Simon

October 19, 2017

1 Mouse Data: Per Sample QC outputs

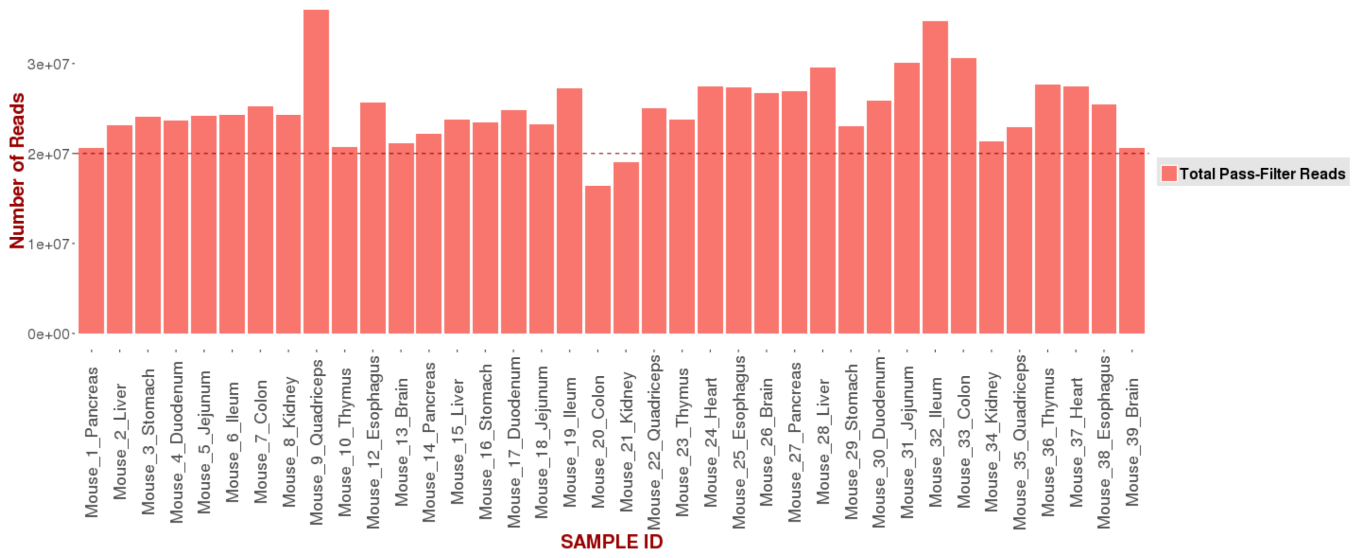

Figure A: Read counts for each mouse sample. The dashed line marks the target of 20 million reads.

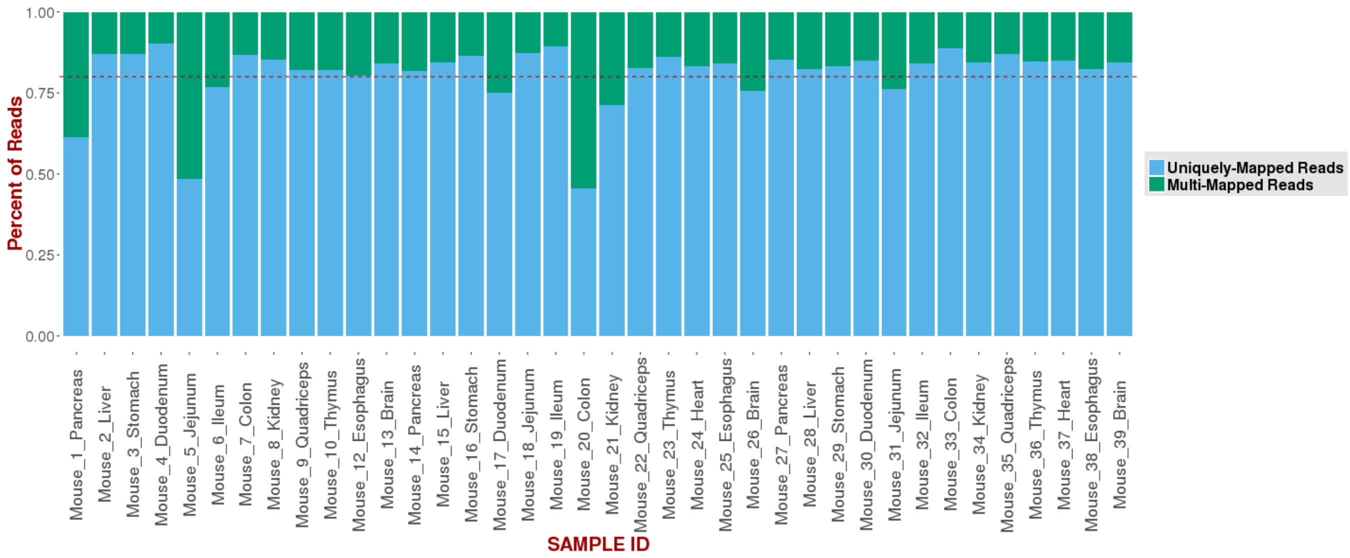

Figure B: Percentage of uniquely and multi-mapping reads for each mouse sample. The dashed line marks the 80% threshold for uniquely mapped reads.

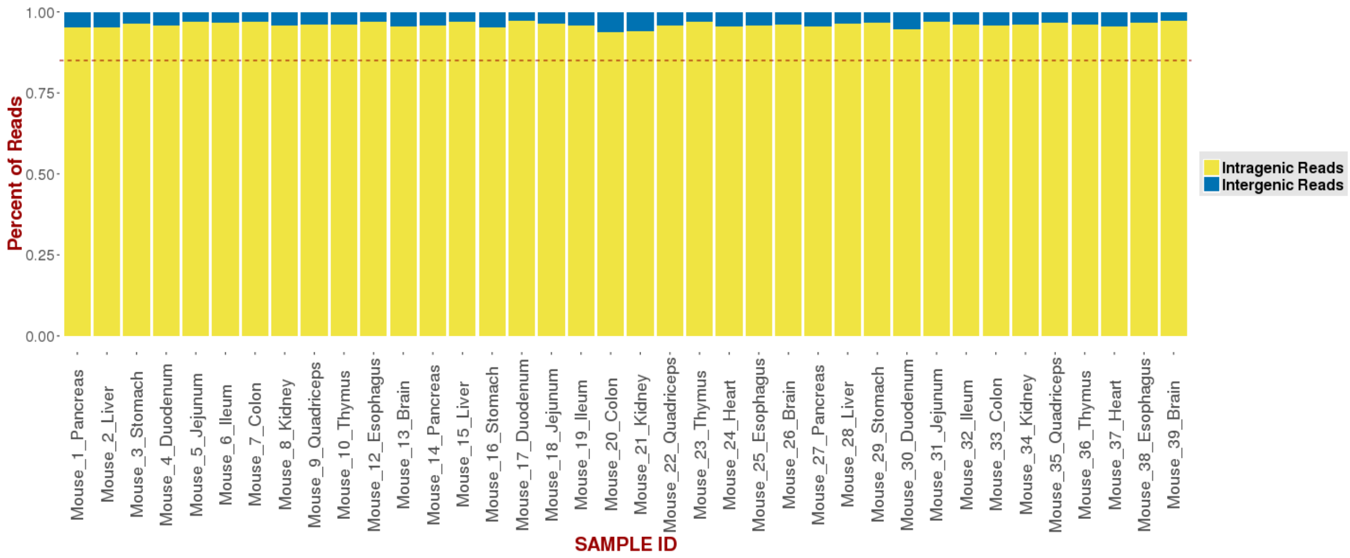

Figure C: The percentage of reads mapping to inter- vs. intra-genic regions for each mouse sample. The dashed line marks the 85% threshold for intragenic mapping.

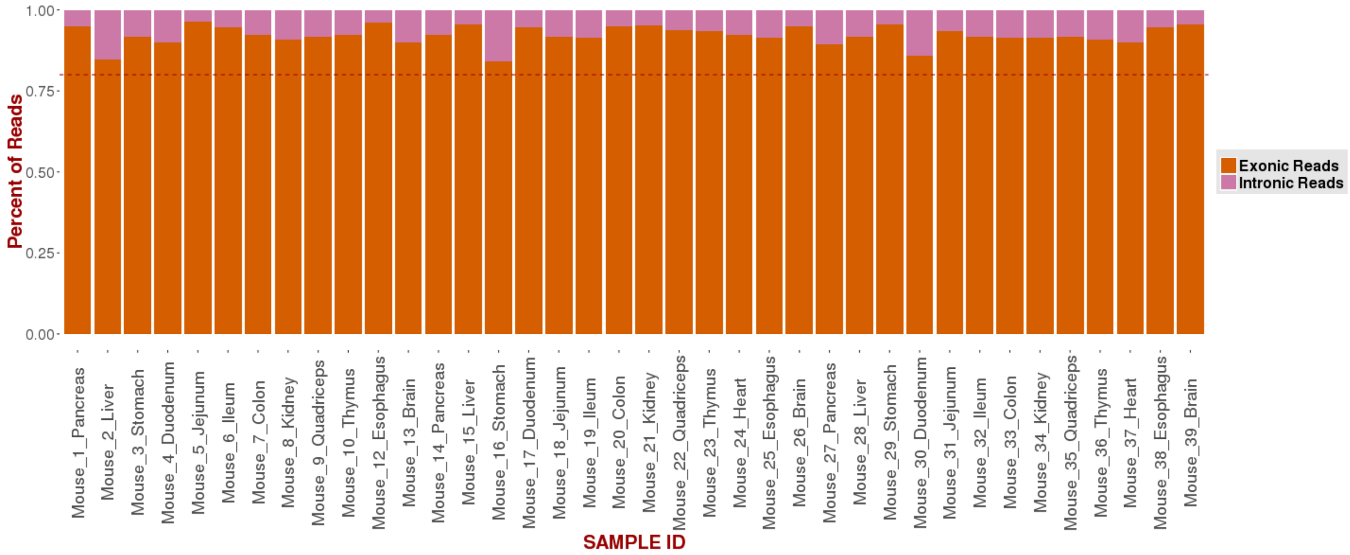

Figure D: The percentage of reads mapping to exonic vs. intronic regions for each mouse sample. The dashed line marks the 80% threshold for exonic mapping.

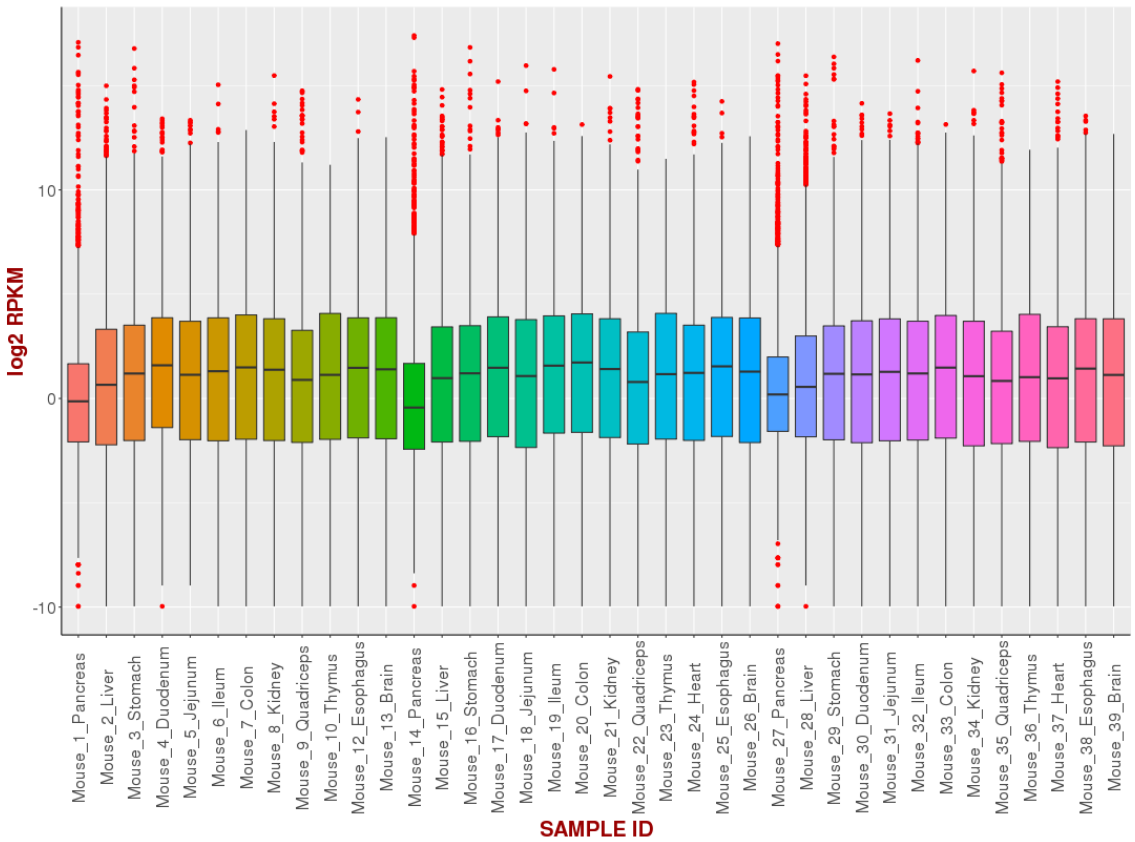

Figure E: Boxplot of the logarithmized RPKM values for each mouse sample.

2 Rat Data: Per Sample QC outputs

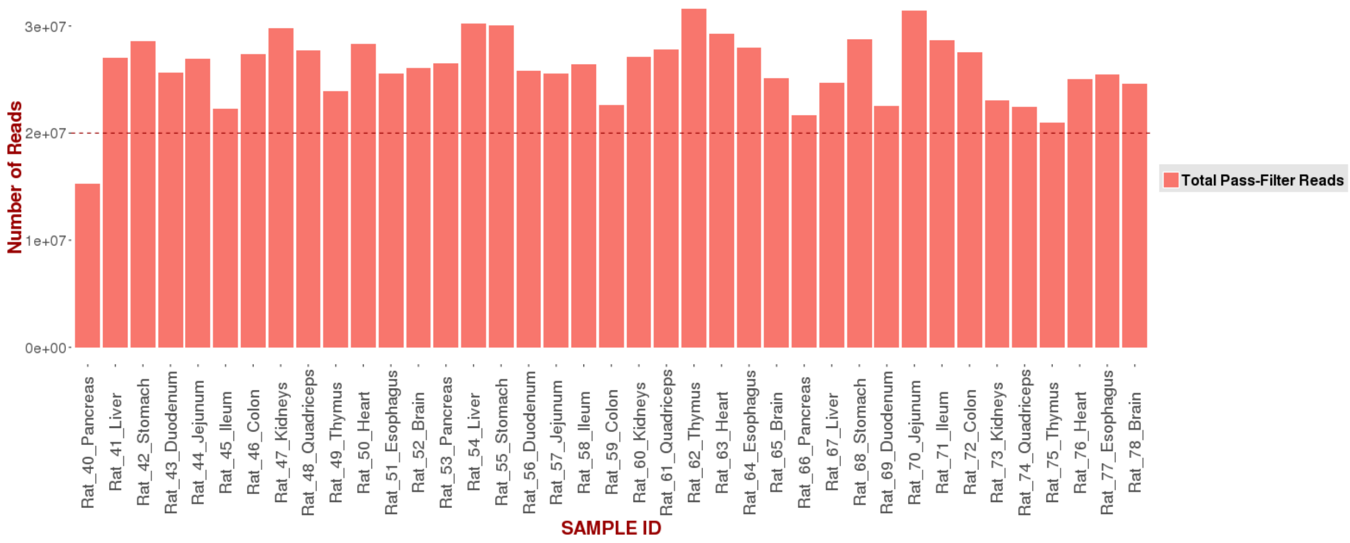

Figure F: Read counts for each rat sample. The dashed line marks the target of 20 million reads.

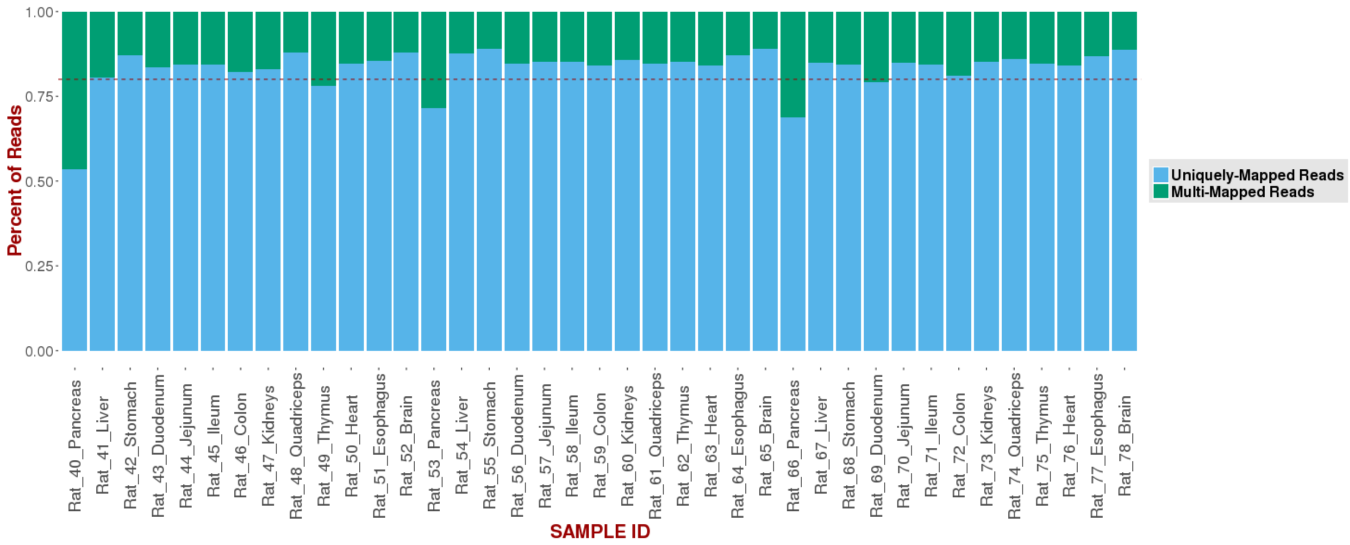

Figure G: Percentage of uniquely and multi-mapping reads for each rat sample. The dashed line marks the 80% threshold for uniquely mapped reads.

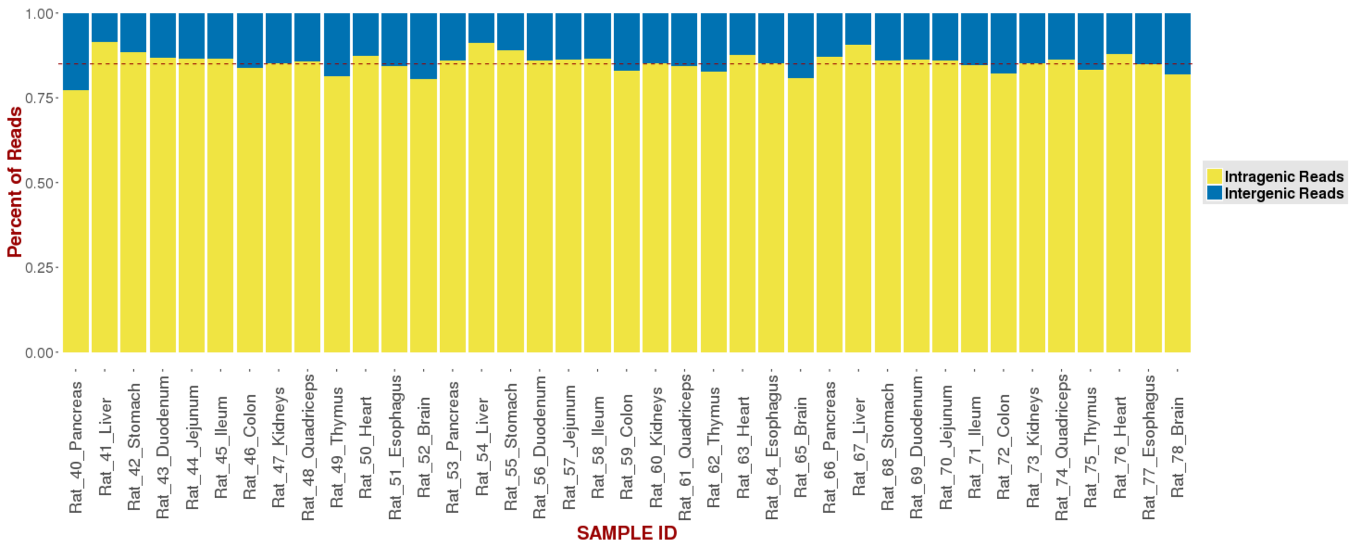

Figure H: The percentage of reads mapping to inter- vs. intra-genic regions for each rat sample. The dashed line marks the 85% threshold for intragenic mapping.

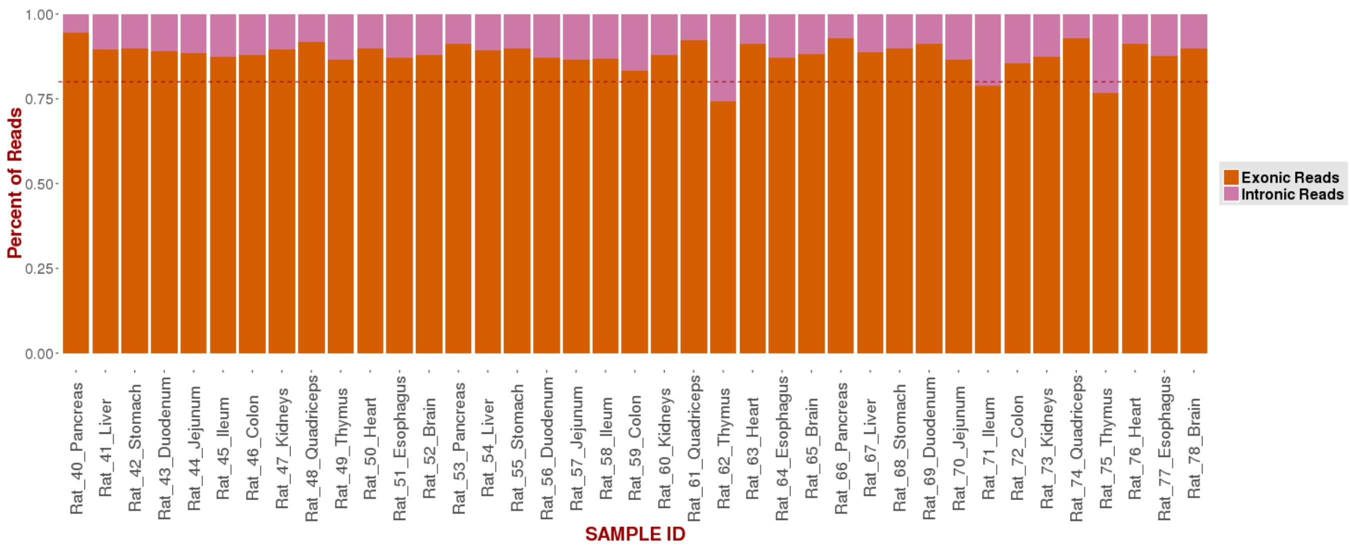

Figure I: The percentage of reads mapping to exonic vs. intronic regions for each rat sample. The dashed line marks the 80% threshold for exonic mapping.

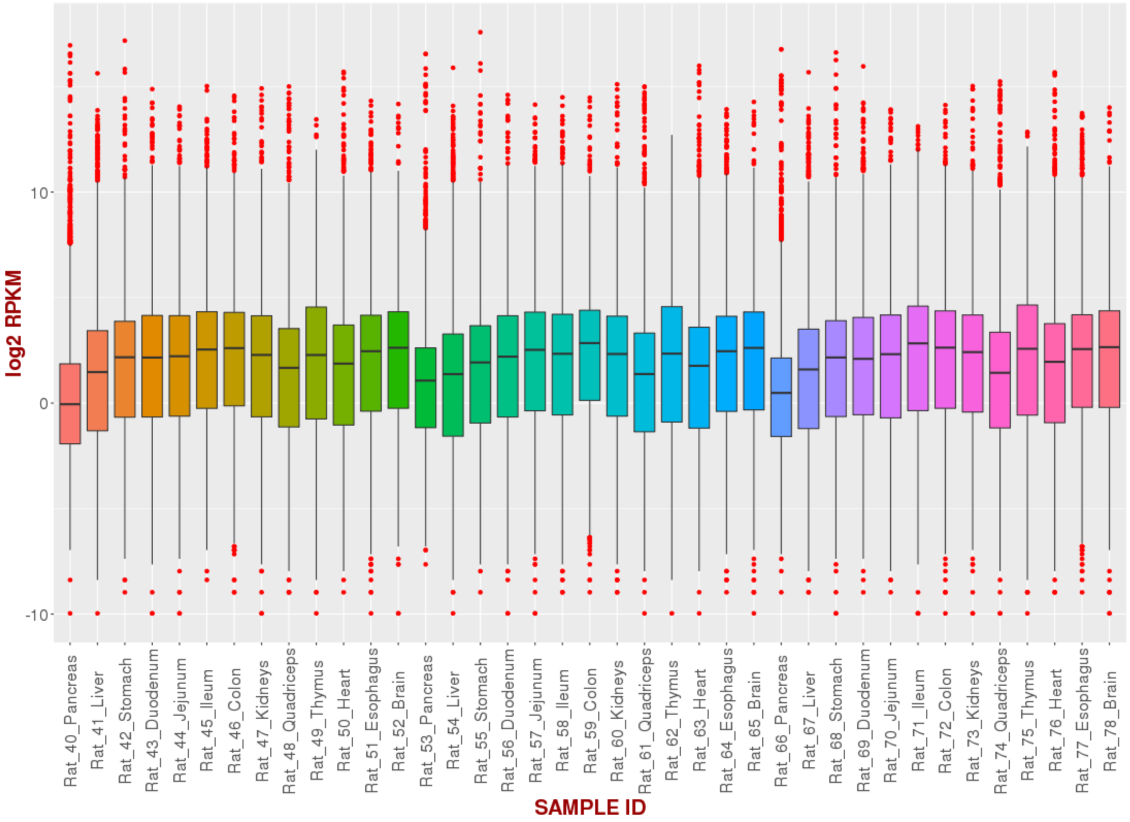

Figure J: Boxplot of the logarithmized RPKM values for each rat sample.
